# Subchronic Exposure to Arsenic Represses the TH/TRβ1-CaMK IV Signaling Pathway in Mouse Cerebellum

**DOI:** 10.3390/ijms17020157

**Published:** 2016-01-26

**Authors:** Huai Guan, Shuangyue Li, Yanjie Guo, Xiaofeng Liu, Yi Yang, Jinqiu Guo, Sheng Li, Cong Zhang, Lixin Shang, Fengyuan Piao

**Affiliations:** 1 Department of Obstetrics and Gynecology, General Hospital of Beijing Military Command, Beijing 100700, China; greeneve@163.com; 2 Department of Obstetrics and Gynecology, No. 210 Hospital of PLA, Dalian 116021, China; liuxiaofengdl210@163.com; 3 Department of Occupational and Environmental Health, Dalian Medical University, Dalian 116044, China; lsy236@163.com; 4 Department of Microecology, Dalian Medical University, Dalian 116044, China; guoyanjie829@163.com; 5 Bayi Brain Diseases Hospital Affiliated to General Hospital of Beijing Military Command, Beijing 100700, China; yyeditor@163.com; 6 Editorial Department, Journal of Dalian Medical University, Dalian 116044, China; green15015@163.com; 7 Department of Biochemistry and Molecular Biology, Dalian Medical University, Dalian 116044, China; lisheng_1996@163.com; 8 School of Public Health, Dalian Medical University, Dalian 116044, China; Congzhang1203@hotmail.com

**Keywords:** arsenic, thyroid hormone receptor, CaMK IV, cerebellum, retinoid X receptor

## Abstract

We previously reported that arsenic (As) impaired learning and memory by down-regulating calmodulin-dependent protein kinase IV (CaMK IV) in mouse cerebellum. It has been documented that the thyroid hormone receptor (TR)/retinoid X receptor (RXR) heterodimer and thyroid hormone (TH) may be involved in the regulation of CaMK IV. To investigate whether As affects the TR/RXR heterodimer and TH, we determined As concentration in serum and cerebellum, 3,5,3’-triiodothyronine (T3) and thyroxin (T4) levels in serum, and expression of CaMK IV, TR and RXR in cerebellum of mice exposed to As. Cognition function was examined by the step-down passive avoidance task and Morris water maze (MWM) tests. Morphology of the cerebellum was observed by H*e*matoxylin-Eosin staining under light microscope. Our results showed that the concentrations of As in the serum and cerebellum of mice both increased with increasing As-exposure level. A significant positive correlation was found between the two processes. Adeficit in learning and memory was found in the exposed mice. Abnormal morphologic changes of Purkinje cells were observed in cerebellum of the exposed mice. Moreover, the cerebellar expressions of CaMK IV protein and the TRβ gene, and TRβ1 protein were significantly lower in As-exposed mice than those in controls. Subchronic exposure to As appears to increase its level in serum and cerebella of mice, impairing learning and memory and down-regulating expression of TRβ1 as well as down-stream CaMK IV. It is also suggested that the increased As may be responsible for down-regulation of TRβ1 and CaMK IV in cerebellum and that the down-regulated TRβ1 may be involved in As-induced impairment of learning and memory via inhibiting CaMK IV and its down-stream pathway.

## 1. Introduction

Arsenic (As) is a common and pervasive contaminant on the earth and several million people are exposed to As in varying concentrations dependent on region. In contaminated areas, the As concentration in drinking water or groundwater often ranges from 0.25 to 2.1 ppm and even reaches >4.0 ppm in some severely contaminated areas of China [1,2,3,4]. Many health problems have been associated with As exposure and the neurological system is considered as one of the major targets. Epidemiological studies showed that As exposure resulted in a dose-dependent reduction in intellectual function in children [5,6]. Experimental studies demonstrated that As impaired learning ability and neural behavior in rodents at environmental relevant levels [7,8]. Our previous study also found that learning and memory were undermined in mice subchronically exposed to As [9]. The above findings indicated that As-induced neurotoxicity may be involved in morphological and functional abnormalities of the central nervous system. Moreover, most of the studies focused primarily on adverse effects of As on the cerebral cortex and hippocampus. Recently, the toxic effects of As on the cerebellum gradually attracted attention. Ding *et al.* [10] demonstrated that drinking As-contained water (4 ppm) during gestation and lactation As could adversely affect cerebellar development in mice. Liu *et al.* [11] found that As treatment (7.5 mg/kg/d, 16 weeks) could induce apoptosis in cerebellar granule neurons. Our previous study showed that As exposure via drinking water (1 ppm, 2 ppm) resulted in damage to cerebellar neurons including Purkinje cells [12], and the same changes were also reported by Kato *et al.* [13]. In addition, it has been reported that people using water contaminated with high-level As (4.5 mg /L) particularly experienced cerebella symptoms [14]. The above findings indicated that the cerebellum may be a target of As-induced neurotoxicity.

Some reports in the literature have documented that the cerebellum contributes to motor learning, and that cerebellar long-term depression (LTD) plays a central role in motor learning [15]. Cerebellar learning and memory requires the activation of the transcription factor CAMP-responsive element binding protein (CREB) [16,17,18]. And signaling by the Ca^2+^/calmodulin-dependent protein kinase IV (CaMK IV) cascade has been implicated in CREB activation mediated LTD [19]. CaMK IV inhibition was reported to attenuate LTD, and mice with CaMK IV defects exhibited impaired neuronal CREB phosphorylation and Ca^2+^/CREB-dependent gene expression, as well as neurological deficits [16,19,20,21]. Our previous study found that subchronic exposure to As significantly down-regulated gene expression of CaMK IV, CREB and memory proteins, c-Fos and Jun B, in mouse cerebellar tissue [9]. The above findings indicated that there might be an association between the down-regulated expression of CaMK IV in the cerebellum and the As-induced deficit of learning and memory, but the mechanism of As-down-regulated expression of CaMK IV in the cerebellum remained unclear.

Transcriptional activation of CaMK IV requires the binding of thyroid hormone (TH) and the thyroid hormone receptor (TR)/retinoid X receptor (RXR) heterodimer, and subsequent binding of this complex to the thyroid hormone-responsive element (TRE) [22,23,24]. TRα1, TRβ1 and TRβ2, TR isoforms, which could bind to TH and target DNA sequence TREs in the CaMK IV gene 5′-flanking region as heterodimers with RXR [23]. Then, the transcription of the CaMK IV gene would be activated. Morte *et al.* [24] reported that CaMK IV was regulated directly by 3,5,3′-triiodothyronine (T3) in primary cultured neurons, and that the CaMK IV protein was also induced by TH. It was also found that As could disrupt TR-mediated gene regulation in rat pituitary GH3 cells [25]. Therefore, we speculated that toxic effect of As on TR/RXR heterodimer and TH may be involved in the down-regulated expression of CaMK IV in mice exposed to As.

In the present study, mice were subchronically exposed to 1, 2, and 4 mg/L arsenic trioxide (As_2_O_3_) via As-containing drinking water. Following exposure, cognition function was examined by step-down passive avoidance task and Morris water maze (MWM) tests. Morphology of cerebellum was observed under the light microscope. The concentrations of As in serum and cerebellar tissue were determined by inductively coupled plasma-mass spectrometry (ICP-MS). The serum levels of TH, T3 and thyroxine (T4), were measured by radioimmunoassay (RIA). The expressions of genes related to theTR/RXR heterodimer in cerebellum were analyzed by real time RT-PCR. Then, the expressions of CaMK IV, TR and RXR proteins in cerebellum were further examined by Western blot. This study aimed at investigating the influence of subchronic exposure to As on the CaMK IV, TR/RXR heterodimer in cerebellar tissue and TH in serum of mice, and exploring the molecular mechanism of As-induced neurotoxicity via inhibition of the TH/TRβ1-CaMK IV signaling pathway.

## 2. Results

### 2.1. As Concentration in Mouse Serum and Cerebellum

**Figure 1:**
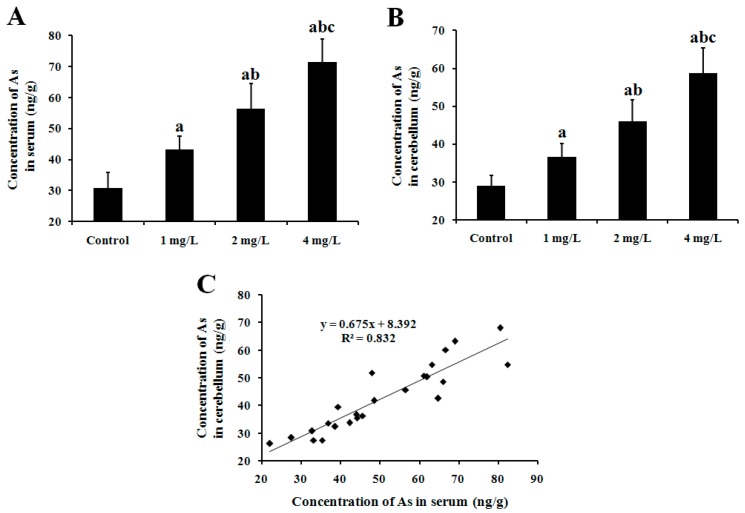
Concentration of As in serum (**A**) and cerebellum (**B**) of mice. Adult male mice exposed to 0, 1, 2, and 4 mg/L As_2_O_3_ in drinking water for 60 days. After the treatment, the concentration of As in serum and cerebellum of mice was determined by ICP-MS. Data obtained from six separate analyses are expressed as mean ± SD (*n* = 6 for each group); (**A**,**B**) ^a^ *p* < 0.05, significantly different compared with the control group; ^b^ *p* < 0.05, significantly different compared with the 1 mg/L As_2_O_3_-treated group; ^c^ *p* < 0.05, significantly different compared with the 2 mg/L As_2_O_3_-treated group; (**C**) Correlation relationship between cerebellar and serum concentrations of As analyzed by Pearson correlation analysis. *R*^2^ = 0.822, *p* < 0.01. Regression equation was *y* = 0.675*x* + 8.392.

During the experimental period, the general appearance and physical condition of mice were observed. There were no significant changes in body weight and daily water consumption among groups. Moreover, the obvious clinical signs of toxicity such as irritability and dysphoria *etc.* were not found in the exposed mice. After exposure to As_2_O_3_ for 60 days, As concentrations in mouse serum and cerebellum were detected by ICP-MS. As shown in Figure 1, the concentration of As in serum was 30.81, 43.23, 56.43, and 71.59 ng/g, respectively, in the control (0), 1, 2 and 4 mg/L As_2_O_3_-treated groups. Compared with the control, the serum As concentrations in the three As-exposed groups increased significantly (*p* < 0.05) in a dose-dependent manner (Figure 1A). The concentration of As in cerebellum was 28.94, 36.49, 46.02 and 58.54 ng/g accordingly in the four groups. Similarly, cerebellar As concentration significantly increased in a dose-dependent manner (*p* < 0.05) (Figure 1B). Further analysis showed a significantly positive correlation between serum and cerebellar As concentrations (*p* < 0.05), with the correlation coefficient (*R*^2^) being 0.822 by linear correlation assay (Figure 1C).

### 2.2. Morphology of the Cerebellum

**Figure 2:**
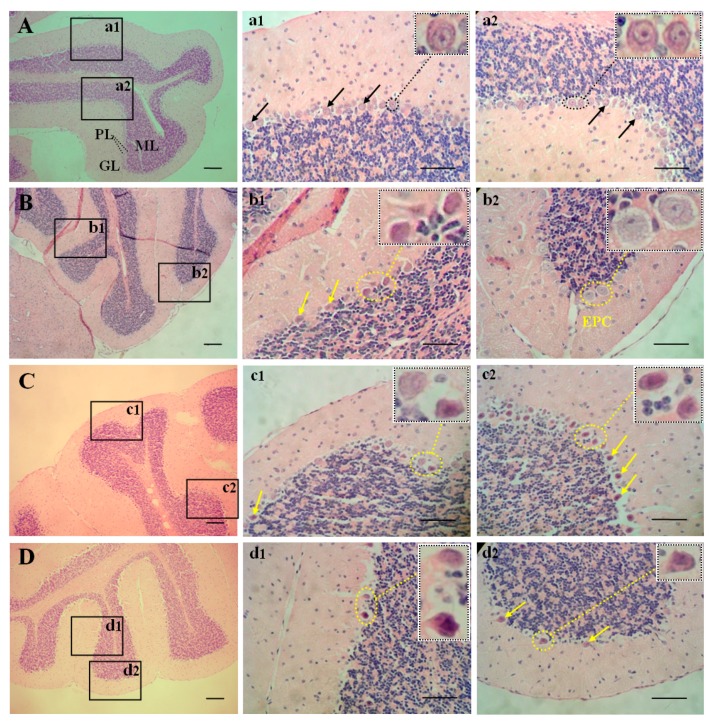
Effect of As exposure on cerebellar morphology of mice. Adult male mice exposed to 0, 1, 2, and 4 mg/L As_2_O_3_ in drinking water for 60 days. After treatment, brain sections of mice were stained with hemotoxylin and eosin. Morphology of cerebellum was observed under light microscope. (**A**) a1, a2: control; (**B**) b1, b2: 1 mg/L As_2_O_3_-treated group; (**C**) c1, c2: 2 mg/L As_2_O_3_-treated group; (**D**) d1, d2: 2 mg/L As_2_O_3_-treated group. ML: molecular layer; PL: Purkinje cell layer; GL: granule cell layer; EPC: edematous Purkinje cell. Black arrow: normal Purkinje cell; yellow arrow: shrinked Purkinje cell. Small rectangular pictures surrounded by dotted line in a1-d1, and a2-d2 were the enlarged images of corresponding oval pictures surrounded by dotted lines. Scale bars, 50 µm.

Brain sections from all four groups showed the typical three layers of cerebellar cortex: the molecular layer, the Purkinje cell layer, and the granule cell layer from the surface to the inside (Figure 2A–D). It is worth noting that morphology of the Purkinje cell layer appeared obviously different among groups. In control mice, Purkinje cells were distributed side by side in one layer. The cytoplasm was stained uniformly, nuclei was large, and karyolemma and nucleolus were clearly shown (Figure 2A,a1,a2). In the 1 mg/L As_2_O_3_-treated group, some Purkinje cells showed reduction in cell body, accompanied by cytoplasmic concentration and deeply stained nuclei (Figure 2B,b1). Some other Purkinje cells showed manifestations of edema, increased cell body, and lightly stained cytoplasm and nuclei (Figure 2B,b2). In the 2 mg/L As_2_O_3_-treated group, cell body shrinkage was observed in most Purkinje cells, accompanied by deeply stained cytoplasm and nuclei, and obscure nuclear structures (Figure 2C,c1,c2). In the 4 mg/L As_2_O_3_-treated group, beside cell shrinkage, the number of Purkinje cells was also reduced (Figure 2D,d1,d2).

### 2.3. Performance of the Behavioral Tests

#### 2.3.1. The Step-Down Passive Avoidance Task

**Figure 3:**
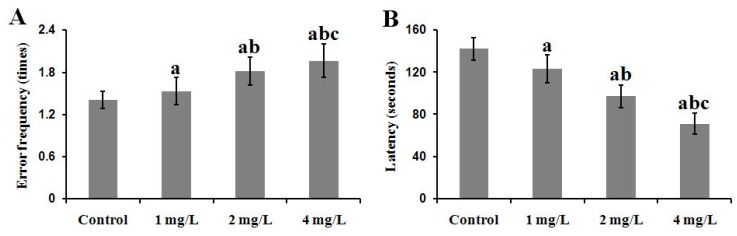
Effect of As exposure on state-dependent learning and memory abilities of mice. Adult male mice exposed to 0, 1, 2, and 4 mg/L As_2_O_3_ in drinking water for 60 days. After the treatment, the step-down passive avoidance task was performed to analyze the state-dependent learning and memory abilities of mice. Data obtained from twelve separate analyses are expressed as mean ± SD *n* = 12 for each group). (**A**) Error frequency of step-down (times); (**B**) Latency to descend from the platform (seconds ). ^a^ *p* < 0.05 significantly different compared with the control group; ^b^ *p* < 0.05, significantly different compared with the 1 mg/L As_2_O_3_-treated group; ^c^ *p* < 0.05 significantly different compared with the 2 mg/L As_2_O_3_-treated group.

In the step-down passive avoidance task, the error frequency of the three exposed groups to step down from a platform after foot-stock significantly increased compared with the control in a dose-dependent manner on the test day (*p* < 0.05) (Figure 3A). The latency of the three exposed groups to descend from the platform 24 h after the training significantly decreased compared with the control in a dose-dependent manner (*p* < 0.05) (Figure 3B). These findings indicated that As exposure resulted in impairment of state-dependent learning and memory of mouse.

#### 2.3.2. The MWM Tests

**Figure 4:**
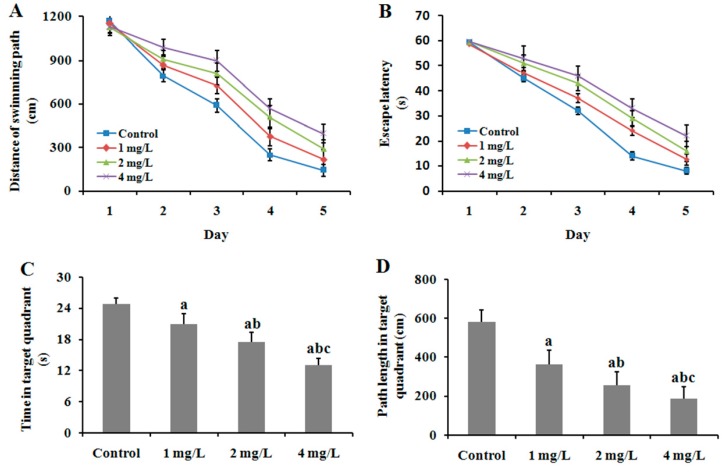
Effect of As exposure on learning and memory abilities of mice. Adult male mice exposed to 0, 1, 2, and 4 mg/L As_2_O_3_ in drinking water for 60 days. After the treatment, the MWM tests were performed to analyze the learning and memory abilities of mice. Data obtained from twelve separate analyses are expressed as mean ± SD (*n* = 12 for each group). (**A**) Distance of swimming path (cm) in the hidden platform test; (**B**) Escape latency to reach the platform (s) in the hidden platform test; (**C**) Time spent in target quadrant (s) in the probe test; (**D**) Path length in target quadrant (cm) in probe test. ^a^ *p* < 0.05 significantly different compared with the control group; ^b^ *p* < 0.05, significantly different compared with the 1 mg/L As_2_O_3_-treated group; ^c^ *p* < 0.05 significantly different compared with the 2 mg/L As_2_O_3_-treated group.

In the hidden platform test, as the training days increased, the average swimming distances and escape latencies for the four groups showed a decreasing trend (Figure 4A,B), indicating that the mice in each group were able to learn to locate the hidden platform to some extent. For swimming distance, there were significant effects of group (*F* = 26.24, *p* < 0.01) and training day (*F* = 33041.26, *p* < 0.01), with a significant interaction between group and training day (*F* = 279.19, *p* < 0.01) (Figure 4A). Similarly, for escape latency, there were significant effects of group (*F* = 62.15, *p* < 0.01) and training day (*F* = 5689.51, *p* < 0.01), with a significant interaction between group and training day (*F* = 44.69, *p* < 0.01) (Figure 4B). These results suggest that both As treatment and training significantly influenced the performance of mouse in the hidden platform test. In addition, the effect of As treatment on performance increased with time. Comparison within groups by least significant difference (LSD) test revealed that the three As-treatment groups showed longer distances and latencies to find the platform than the control group (*p* < 0.05). Moreover, there was a significant difference in distance and latency between 1 and 2 mg/L As_2_O_3_-treated groups, as well as between 2 and 4 mg/L As_2_O_3_-treated groups. These results suggest that As-induced impairment of spatial learning ability in mouse was aggravated with treatment dose increase. In the probe test, the time spent in the target quadrant in the three exposed groups was significantly shorter compared with controls in a dose-dependent manner (*p* < 0.05) (Figure 4C). The same effect was also observed in path length of swimming in the target quadrant (*p* < 0.05) and decreased (Figure 4D). These findings indicate that As exposure results in impairment of spatial memory retention in mouse.

### 2.4. Protein Expression of CaMK IV in Mouse Cerebellum

**Figure 5:**
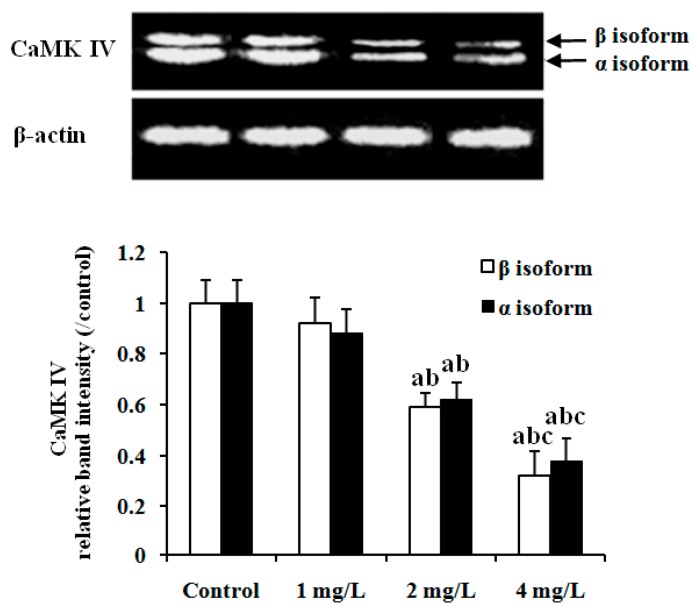
Effect of As exposure on protein expression of CaMK IV in cerebellar tissue of mice. Adult male mice exposed to 0, 1, 2, and 4 mg/L As_2_O_3_ in drinking water for 60 days. After the treatment, the expression of CaMKIV protein was analyzed by western blot. The relative CaMK IV protein abundance was determined by the ratio of sample to β-actin. Data obtained from six separate analyses are expressed as mean ± SD (*n* = 6 for each group). The double bands corresponded to α and β isoforms of CaMK IV protein, respectively. ^a^ *p* < 0.05 significantly different compared with the control group; ^b^ *p* < 0.05 significantly different compared with the 1 mg/L As_2_O_3_-treated group; ^c^ *p* < 0.05 significantly different compared with the 2 mg/L As_2_O_3_-treated group.

Protein expression of CaMK IV in mouse cerebellum was detected by Western blot. As shown in Figure 5, the double bands corresponded to α and β isoforms of CaMK IV protein respectively. Expression of both α and β isoforms of CaMK IV significantly decreased in the 2 and 4 mg/L As_2_O_3_-treated groups compared to 1 mg/L As_2_O_3_-treatedor control groups (*p* < 0.05). Moreover, the expression of these two isoforms in the 4 mg/L As_2_O_3_-treated group was also significantly lower than that in the 2 mg/L As_2_O_3_-treated group (*p* < 0.05).

### 2.5. Gene and Protein Expression of TR in Mouse Cerebellum

**Figure 6:**
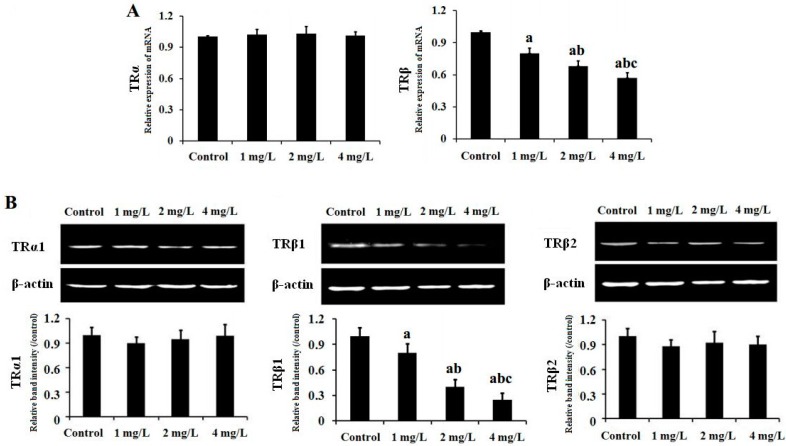
Effect of As exposure on gene (**A**) and protein (**B**) expression of TR in cerebellar tissue of mice. Adult male mice exposed to 0, 1, 2, and 4 mg/L As_2_O_3_ in drinking water for 60 days. After the treatment, the expression of the TR gene and protein (three isoforms,TRα1, TRβ1 and TRβ2) was analyzed by real time RT-PCR and western blot, respectively. The relative abundance of TR mRNA and protein was determined by the ratio of sample to β-actin. Data obtained from six separate analyses are expressed as mean ± SD (*n* = 6 for each group). ^a^ *p* < 0.05 significantly different compared with the control group; ^b^ *p* < 0.05 significantly different compared with the 1 mg/L As_2_O_3_-treated group; ^c^ *p* < 0.05 significantly different compared with the 2 mg/L As_2_O_3_-treated group.

The mRNA expression of TR in mouse cerebellum was detected by real time RT-PCR. As shown in Figure 6A, the expression of TRβ mRNA was significantly lower in the treated groups than that in controls (*p* < 0.05) and decreased in a dose-dependent manner. However, there was no significant difference in mRNA expression of TRα among the four groups (*p* > 0.05).

TR protein expression in mouse cerebellum was then detected by Western blot. As shown in Figure 6B, TRβ1 protein level was significantly lower in the three As-exposed groups than in the control (*p* < 0.05) and decreased in a dose-dependent manner. However, there was no significant change in protein expression of TRα1 or TRβ2 among the four groups (*p* > 0.05).

### 2.6. Gene and Protein Expression of RXR in Mouse Cerebellum

**Figure 7:**
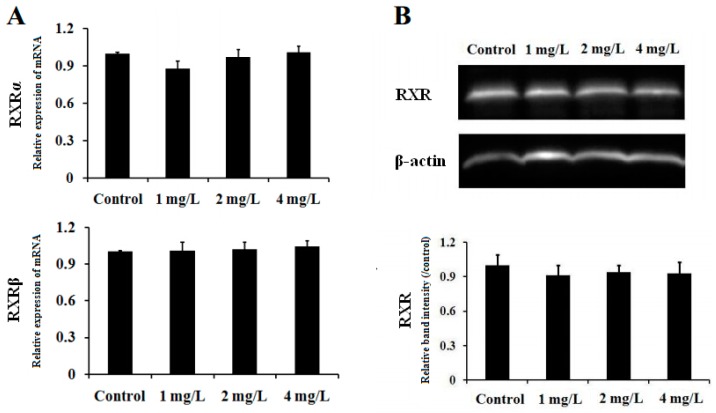
Effect of As exposure on gene (**A**) and protein (**B**) expression of RXR in cerebellar tissue of mice. Adult male mice exposed to 0, 1, 2, and 4 mg/L As_2_O_3_ in drinking water for 60 days. After the treatment, the expression of RXR gene and protein was analyzed by real time RT-PCR and western blot, respectively. The relative abundance of RXR mRNA and protein was determined by the ratio of sample to β-actin. Data obtained from six separate analyses are expressed as mean ± SD (*n* = 6 for each group).

As a partner together with TR to form heterodimers, RXR was also determined in gene and protein levels by real time RT-PCR and Western blot. As shown in Figure 7A, there was no significant change in mRNA expressions of both α and β isoforms among the four groups (*p* > 0.05). Moreover, no significant change in RXR protein expression was observed among As_2_O_3_-treated and control groups (*p* > 0.05), Figure 7B in accordance with the results at the mRNA level.

### 2.7. T3 and T4 Levels in Serum

**Figure 8:**
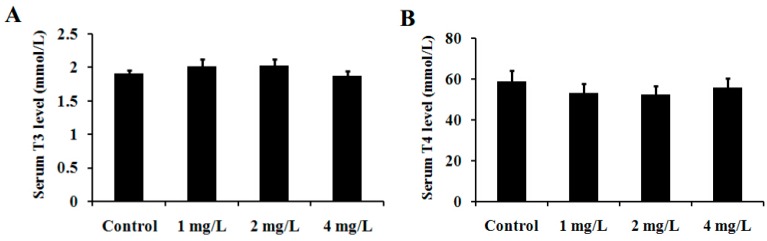
Effect of As exposure on serum concentrations of T3 (**A**) and T4 (**B**) of mice. Adult male mice exposed to 0, 1, 2, and 4 mg/L As_2_O_3_ in drinking water for 60 days. After the treatment, the concentrations of T3 and T4 in mice serum were determined by RIA. Data obtained from six separate analyses are expressed as mean ± SD (*n* = 6 for each group).

T3 and T4 levels in serum were determined by RIA and the results are presented in Figure 8. As shown, the serum T3 levels in control, 1, 2 and 4 mg/L As_2_O_3_-treated groups were (1.91 ± 0.66), (2.01 ± 0.92), (2.03 ± 0.90) and (1.88 ± 0.81) mmol/L, respectively; while the serum T4 levels in the four groups were (59.04 ± 7.2), (58.62 ± 6.5), (59.77 ± 5.9) and (58.54 ± 6.8) mmol/L, respectively. No significant difference in serum levels of the two thyroid hormones was observed among the four groups (*p* > 0.05).

## 3. Discussion

Epidemiological studies have demonstrated that As causes impairments of learning [26] and deterioration in pattern memory and switching attention in human [27]. In animal experiments, exposure to As led to delay in learning acquisition [28], alterations in locomotor behavior and deficits in spatial learning paradigms [29,30]. In the present study, mice exposed to As showed higher error frequencies and shorter latency than the control in the step-down passive avoidance task (Figure 3A,B). In the MWM tests, the treated mice required a longer swimming distance and escape latency period to find the hidden platform (Figure 4A,B), consistent with the above studies. These results suggest that As exposure impaired state- and spatial-dependent learning and memory abilities in mice. However, the mechanism underlying how to induce the neurotoxicity is still unclear. It is well known that learning and memory are cognitive functions associated mainly with the hippocampus and cerebral cortex. However, during the past decade, studies in animals and man have implicated that the cerebellum is essential in the processing of signals not only for motor function but also for perception, cognition, and emotion [31,32,33]. Some neurotoxic substances induced neurotoxicity in the cerebellum in experimental models; in particular, toxic effects of As on cerebellum biology has been investigated. In our previous study, we found that the expression of some important genes associated with learning and memory were disturbed in the cerebellum of mice exposed to As, indicating cerebellar involvement in learning and memory deficit induced by As.

It was reported that As was dose-dependently accumulated in various organs and tissues. Moreover, It was shown in animal experiments that As could pass through the blood-brain barrier and invade the brain parenchyma [8,11]. In our study, the concentration of As in serum and the cerebellum of mice was determined. Our results showed that the concentrations of As in serum and cerebellum were significantly higher in exposed mice than those in controls and increased in a dose-dependent manner (Figure 1A,B). Moreover, there was a noticeable positive correlation between the serum and cerebellar As concentrations in experimental groups (Figure 1C). These results indicate that subchronic exposure to As increases its level in serum and cerebella of mice, and the higher the level of As in the blood, the higher the concentration becomes in the cerebellum. To examine adverse effect of As on the cerebellum, cerebellar morphology was also observed in our study. Our results showed that exposure to low levels of As (1 mg/L As_2_O_3_) induced shrinkage and edema in Purkinje cells (Figure 2B,b1,b2). Exposure to middle to high levels of As (2 and 4 mg/L As_2_O_3_) induced aggravated shrinkage in Purkinje cells (Figure 2C,c1,c2,D,d1,d2). High levels As (4 mg/L As_2_O_3_) even resulted in loss of Purkinje cells (Figure 2D,d1,d2). These results indicate that the accumulated As can lead to abnormal changes in cerebellar morphology.

It has been demonstrated that cerebellar LTD plays a central role in motor learning [15], and CREB activation is essential to the maintenance of LTD. CaMK IV-dependent activation of CREB is a critical step for mediating many genes involved in the process of neuronal plasticity [16]. CaMK IV-deficient mice showed defects in a late phase of long-term depression in cerebellar Purkinje neurons [21]. CaMK IV-deficient mice also exhibit impaired neuronal CREB phosphorylation and Ca^2+^/CREB-dependent gene expression in neurons [21]. The above studies indicate that CaMK IV contributes to activating CREB and plays a critical role in the induction of LTD in the cerebellum.In our previous study, we found that subchronic exposure to As significantly down-regulated expression of the CaMK IV gene and its protein in the cerebellum of mice. The present results also showed that α and β isoform expression of the CaMK IV protein was significantly down-regulated in the cerebellum of mice exposed to As compared to controls (Figure 5), supporting our previous study. The results indicated that As repressed CaMK IV and the CaMK IV-regulated pathway in the cerebellum.

*In situ* hybridization studies indicated that CaMK IV is regulated by TH in a time- and concentration-dependent manner in the developing rat brain [34]. In cultured rat fetal telencephalon cells, CaMK IV was induced by addition of T3 [35]. Liu and Brent [23] have demonstrated regulation of endogenous CaMK IV by T3 in embryonic stem cells differentiated into neurons. Moreover, TRE has been identified in the CaMK IV gene promoter, conferring T3 responsiveness and binding TR [23]. The studies indicate that TH regulates CaMK IV expression through binding to the TR. THs are synthesized by the thyroid gland, including T4 and T3. T3 is an active TH ligand and is produced when type I or type II deiodinase removes the specific outer ring iodine of T4 [25]. TR normally partners with the RXR to form heterodimers that act as a functional transcription factor, binding to the TRE of TH-inducible genes to activate or repress transcription of target genes [36]. RXRs are T3 receptor auxiliary proteins and have been shown to enhance TR binding to its cognate TREs, and augment T3-mediated transcriptional activation [37]. The data suggests that the TR/RXR heterodimer may be a physiologically important receptor complex in T3-stimulated gene expression. Therefore, we were interested in whether As down-regulates expression of CaMK IV in the cerebellum via affecting expression of TR and RXR or the level of TH. In the present study, subchronic exposure to As significantly decreased mRNA expression of TRβ in the cerebellum of mice (Figure 6A). This result was further confirmed by Western blot analysis (Figure 6B). Moreover, the data (not shown) from previous GeneChip analyses also supported our result. However, there were no significant differences in the serum levels of T3 and T4 (Figure 8A,B) or the mRNA expression of cerebellar TRα, RXRα and RXRβ (Figure 7A) among the groups exposed to As and controls. These results indicate that subchronic exposure to As down-regulates expression of the TRβ gene in the cerebellum of mice. Experimental studies indicate that As affects the levels of thyroid hormones in the exposed animals. Mohanta *et al.* (2014) [38] reported that thyroid hormones were reduced in guinea pigs exposed to As. On the contrary, Sun *et al.* [39], reported that the thyroxine level in zebrafish was significantly elevated by As. These studies indicated that many factors such as species, dose, and exposure period *etc.* may affect the toxic effects of As in thyroid function; thus further study to confirm the effects of As on thyroid hormones are necessary.

TRs are encoded by two different genes, TRa and TRβ, and each gene has several alternative mRNA splicing products [37]. The two TRβ isoforms, TRβ1 and TRβ2, differ in their amino termini but are both ligand-dependent transcriptional enhancers. At least three distinct isoforms have been isolated from the human and rat TRα genes. However, only one of these isoforms, TRal, is a ligand-dependent activator of transcription, while the other isoforms are receptor-like molecules that do not bind TH and are not transcriptional activators [40]. Therefore, in the present study, the effect of As on protein expression of the TR isoforms, TRal, TRβ1 and TRβ2 in the cerebellum of mice were further examined by Western blot. We found that subchronic exposure to As significantly decreased expression of cerebellar TRβ1 protein in a dose-dependent manner (Figure 6B). However, the protein expression of cerebellar TRα1 and TRβ2 did not show significant changes in the treated mice (Figure 6B). On the other hand, no significant difference in the expression level of RXR protein was observed between the experimental groups and controls, in accordance with our results at the mRNA level (Figure 7A,B). These results indicate that As down-regulates expression of the cerebellar TRβ1 protein and the repressed TRβ gene may be responsible for the down-regulated cerebellar TRβ1 protein in the treated mice. Studies show that vitamin A deficiency resulted in the reduced expression of TR and RXR, and a concomitant cognition impairment in the brain of mice, hinting at an association between the inhibited TR/RXR signaling and cognitive impairment [41,42]. These results also suggest that down-regulated cerebellar TRβ1 may be involved in As-induced impairment of learning and memory via inhibition of TH-regulated CaMK IV and the down-stream signaling pathway.

## 4. Materials and Methods

### 4.1. Chemicals and Reagents

As_2_O_3_, HNO_3_ and H_2_O_2_ were purchased from Sigma Chemical Company (St. Louis, MO, USA). When used, As_2_O_3_ was weighed and dissolved in dilute NaOH solution, and then the pH of 40 mg/L As_2_O_3_ stock solution was adjusted to 7.2. The primers for real time RT-PCR used in our study were designed by and purchased from Takara Company (Dalian, China). Rabbit anti-mouse polyclonal antibody of CaMK IV was purchased from Cell Signal Technology, Inc. (Beverly, MA, USA); goat anti-mouse polyclonal antibodies of RXR and TRα1 from Abcam (Cambridge, MA, USA); and mouse anti-mouse monoclonal antibodies of TRβ1, TRβ2 and β-actin from Santa Cruz Biotechnology, Inc. (Santa Cruz, CA, USA).

### 4.2. Animals and Treatment

Male Kunming mice, aged 9 weeks and weighing 25.6~32.4 g, were purchased from Experimental Animal Center, Dalian Medical University. The animals were housed in an animal facility maintained in a 12-h (07:00—19:00) light/dark cycle at a constant temperature of 22 ± 3 °C and relative humidity of 55% ± 15% and were fed with common basal pellet diet (As concentration < 0.7 mg/kg) *ad libitum.* After one-week adaptation, the mice were randomly divided into 4 groups, each of 12 animals. Group 1 orally received double-distilled water alone as a control. Groups 2~4 orally received double-distilled water containing As_2_O_3_ at a dose of 1, 2, and 4 mg/L, respectively. After treatment for 60 days, all mice were assigned to undergo behavioral tests. The day after behavioral testing, all mice were killed and different samples were collected according to respective needs. For serum examination and biochemical detection, mouse was killed by isoflurane exposure. Serum was collected by centrifuging clotted samples and then was stored at −80 °C for later analysis of THs and As concentrations. Cerebellum was collected and stored at −80 °C for later detection of As concentrations and genes and proteins of interest. For histopathological studies, mice were deeply anesthetized through intraperitoneal injection of sodium pentobarbital and perfused with physiologic saline and 4% paraformaldehyde, successively. Then, the brain was removed and fixed in 4% paraformaldhyde at 4 °C for 24 h for morphologic analyses. Experiments were performed in accordance with the Animal Guideline of Dalian Medical University and in agreement with the Ethical Committee of Dalian Medical University.

### 4.3. Detection of As Concentration in Mouse Serum and Cerebellum Tissue

As concentrations in mouse serum and cerebellum were detected by ICP-MS according to the procedure previously reported by our study group [43]. Briefly, the samples (0.2 mL serum and (0.2 ± 0.02) g cerebellum in wet weight) were first digested, and then analyzed by ICP-MS, Agilent 7500CE (Agilent Technologies, Palo Alto, CA, USA). He (4.5 mL/L) was used as reaction gas. Ga (1 mg/L, *M*/*z* = 71) was used as an internal standard in this mode instead of common Y or In. The calibration range of As was from 0 to 20 ng/g. The detection limits for As was 0.3 ng/g. In order to ensure the accuracy of ICP-MS analysis, quality control materials (CORTOX, HMB59311, Kauls on Laboratories, Inc., West Caldwell, NJ, USA) were used. Ten percent of samples were tested repeatedly.

### 4.4. Behavioral Testing

Each mouse was handled for 1–2 min per day for 2–3 days prior to beginning the behavioral tests. The behavioral tests consisted of the **s**tep-down passive avoidance task, followed by the Morris water maze (MWM) tests.

#### 4.4.1. Step-down Passive Avoidance Task

The step-down passive avoidance task is used to evaluate state-dependent learning and memory. This test was carried out in the day between 13:00–16:00. The test involves learning not to step down from froma platform in order to avoid a mild foot-shock [44]. The apparatus consisted of a plastic box (30 × 15 × 15 cm^3^) whose floor contains parallel stainless steel bars (0.3 cm diameter) spaced 1 cm apart. A high platform with a diameter of 5 cm and a height of 2.5 cm was placed in the center of the box. Before the test, animals were allowed a 3 min. period for acclimation. In the training session, mice were placed on the platform. Once stepped down on the grid floor, the mice received foot-shocks (36 V) continuously for 180 s, which were delivered to the grid floor by an isolated stimulator (SLY-SRC, Chongqing, China). After receiving foot-shocks, the mouse stepped upon the platform. The frequency of step down onto the grid floor with all four paws within 180 s was recorded. Retention test sessions were carried out 24 h after the training. Mice were placed on the platform and no foot-shock was given. The step-down latency (180 s ceiling) was taken as a measure of avoidance memory. At the end of each test, the surface of the apparatus was thoroughly cleaned to avoid the presence of olfactory cues.

#### 4.4.2. MWM Tests

The MWM is used to evaluate spatial learning and memory, here, learning and memory abilities of mice were examined by the MWM tests. The device is a black circular tank (100 cm diameter and 40 cm height) filled with 30 cm depth of water (25 ± 2) °C. It is geographically divided into 4 equal quadrants (N, S, E, and W). The experiments were performed in a dimly lit room with spatial cues (e.g., circles, squares, or triangles) attached at different points on the walls around the maze. A smart video tracing system (NoldusEthovision^®^ system, version 5, Everett, WA, USA) recorded the performances of mice which were traced on the screen of a computer. The MWM tests consisted of two stages and were conducted in the day between 13:00–16:00. The first stage of the MWM tests was a hidden platform test. A circular transparent escape platform (10 cm diameter and 29 cm height) was submerged 1 cm below the water surface and always located in the center of the N quadrant. The mice were given four trials (once from each starting position) daily with a 10 min inter-trial interval for 5 consecutive training days. Each trial had a maximum duration of 60 s, and a 15 s platform habituation. If mice failed to reach the platform within the maximum allowed time of 60 s, it was gently guided onto the platform and allowed to orient to the distal visual cues. The escape latency and distance to find the hidden platform were collected as the parameters of learning function.The second stage of the MWM tests was a probe test. This test was conducted by removing the escape platform on the 6th day of the MWM tests. Mice, released from the S quadrant, were allowed to navigate freely in the pool for 60 s. The time and path length swimming in the target quadrant, which had previously contained the hidden platform, were recorded as the parameters of memory retention.

### 4.5. Hematoxylin-Eosin (HE) Staining

The fixed brain was trimmed, washed, dehydrated, and embedded in paraffin according to standard protocols. The paraffin block of the brain was cut at 5 µm thickness from the mid portion of tissue along the sagittal plane. After deparaffinization, the sections were stained with hematoxylin and eosin and observed under a light microscope.

### 4.6. Real Time RT-PCR

RNA was extracted from mice cerebellum tissues using Trizol^®^ reagent (Takara, Dalian, China) following the manufacturer’s instructions. The reverse transcription reactions were conducted with Transcriptor First Strand cDNA Synthesis Kit (Roche, Indianapolis, IN, USA). Real time RT-PCR was performed with a SYBR Green PCR kit (Takara, Japan) using a TP800 Real Time PCR Detection System (Takara, Japan). The primers for genes of interest and β-actin are shown in Table 1. The reaction conditions were as follows: an initial denaturation at 95 °C for 5 min, followed by 40 cycles of 95 °C for 30 s, 55 °C for 30 s, and 72 °C for 30 s.

**Table 1:** Primer sequences for real-time RT-PCR.

Gene	Forward Primer	Reverse Primer
*RXRα*	TGAGACATACGTGGAGGCAAACA GAAGAGCCCTTATGATCCCAAAC	GGCCCACTCCACAAGAGTGAA AGATAGCGAAGTCCCGTCCC
*RXRβ*	GCAAACGGCTCTGTGCAATC ATGGCATTCCGGGCGATTT	GCTGGCGCTTGTCCACTGTA GGTTTTGGTTTCTCGAAGCTCA
*TRα*	GACAAGGCCACCGGTTATCACTAC GACAAATGTGGCGGGACCATA	CAGCAGCTGTCATACTTGCAG GA TGGATTAGCCATTCACACTTCTC
*TRβ*	AGCCAGAACCCACGGATGAG AGGACACGAAGTGAGAAGCC	CGATGGGTGCTTGTCCAATG GTGAGGGTTGAAGTTGAGAACA
*β-actin GAPDH*	CATCCGTAAAGACCTCTATGCCAAC AGGTCGGTGTGAACGGATTTG	ATGGAGCCACCGATCCACA TGTAGACCATGTAGTTGAGGTCA

### 4.7. Western Blot

Mice cerebellum tissues were homogenized in ice-cold RIPA Tissue Protein Extraction Reagent (Biyuntian, China) supplemented with 1% proteinase inhibitor mix and incubated at 4 °C for 1 h. After incubation, debris was removed by centrifugation at 12,000× *g* for 15 min at 4 °C, and the lysates were stored at −80 °C until use. The total protein concentration in the lysates was determined using a BCA protein assay kit (Biyuntian, Nantong, China). The proteins (50 µg per lane) were mixed with an equal volume of SDS-PAGE loading buffer, separated by SDS-PAGE under no-reducing conditions using 10% SDS-PAGE gels, and then electrotransferred to a PVDF membrane. The membrane was blocked with blocking buffer containing defatted milk power for 1 h and incubated overnight at 4 °C with the primary antibody (CaMK IV 1:1000; RXR, TRα1 1:1000; TRβ1, TRβ2 1:800; β-actin 1:350). The membrane was washed three times with Tris buffered saline containing 0.05% Tween-20 (TBST) for 10 min and then incubated at 37 °C for 1 h with horseradish peroxidase-conjugated secondary antibody (1:5000). The signals were visualized using an enhanced ECL chemiluminescence kit and quantified densitometrically using a UVP BioSpectrum Multispectral Imaging System (Ultra-Violet Products Ltd., Upland, CA, USA).

### 4.8. RIA

Serum *concentrations of* THs, T3 and T4, were determined by specific Coated-Tube RIA kits (MP Biomedicals, LLC, Santa Ana, CA, USA). Hormone-stripped mouse serum was used for the standard curves of T3 and T4. All the procedures were carried out following the manufacturer’s instructions.

### 4.9. Statistical Analysis

All analysis was performed with the SPSS 17.0 for Windows. Data were presented as the mean ± standard deviation (SD). Indexes of the hidden platform test were analyzed using repeated-measures analysis of variance (ANOVA) followed by a (LSD) test. As levels, THs levels, indexes of other behavioral tests and expressions of interested genes and proteins were analyzed using one-way ANOVA followed by a LSD test. Correlation relationship between cerebellar and serum concentrations of As was analyzed by Pearson correlation analysis. *p*-value <0.05 was deemed statistically significant in all experiments.

## 5. Conclusions

Our results show As accumulation and abnormal morphology in the cerebellum of the treated mice, as well as a deficit in the learning and memory in the As-exposed mice. The cerebellar expression of the CaMK IV protein and TRβ gene and TRβ1 protein was significantly decreased in the mice exposed to As, as compared to that in controls. However, there were no significant differences in the expression of cerebellar *TRα*, *TRβ2*, *RXRα* and *RXRβ* genes among the experimental groups and controls. Our results indicate that increased As exposure levels may be responsible for down-regulation of TRβ1 and CaMK IV in the cerebellum of mice and that down-regulated TRβ1 may be involved in As-induced impairment of learning and memory via inhibition of CaMK IV expression and its down-stream pathway.

Further investigation is required to elucidate the relationship between As-repressed cerebellar expression of CaMK IV and TRβ1 and impaired learning and memory in mice.
